# Oral malignant melanomas and other head and neck neoplasms in Danish dogs - data from the Danish Veterinary Cancer Registry

**DOI:** 10.1186/1751-0147-51-54

**Published:** 2009-12-18

**Authors:** Louise B Brønden, Thomas Eriksen, Annemarie T Kristensen

**Affiliations:** 1 Department of Small Animal Clinical Sciences, Faculty of Life Sciences, University of Copenhagen, Dyrlægevej 16, DK-1870 Frederiksberg C, Denmark

## Abstract

**Background:**

Head and neck cancers (HNC) are relatively common and often very serious diseases in both dogs and humans. Neoplasms originating in the head and neck region are a heterogeneous group. HNC often has an unfavourable prognosis and the proximity of the tissue structures renders extirpation of tumours with sufficient margins almost incompatible with preservation of functionality. In humans oral malignant melanoma (OMM) is extremely rare, but represents a particular challenge since it is highly aggressive as is the canine counterpart, which thus may be of interest as a spontaneous animal model.

**Methods:**

Canine cases entered in the Danish Veterinary Cancer Registry (DVCR) from May 15th 2005 through February 29th 2008 were included in this study. Fisher's exact test was used to compare proportions of HNC in dogs and humans as well as proportions of surgically treated cases of OMM and squamous cell carcinomas (SCC). Also the proportions of benign and malignant neoplasms of different locations in dogs were compared using Fisher's exact test.

**Results:**

A total of 1768 cases of neoplasias (679 malignant, 826 benign, 263 unknown) were submitted. Of all neoplasias HNC accounted for 7.2% (n = 128). Of these, 64 (50%) were malignant and 44 (34%) benign. The most common types of malignant neoplasia were SCC (18; 28% of malignant), OMM (13; 20% of malignant), soft tissue sarcoma (11; 17% of malignant) and adenocarcinoma (5; 11% of malignant). The most common types of benign neoplasms were adenoma (7; 16% of benign), polyps (6; 14% of benign) and fibroma (5; 11% of benign).

**Conclusions:**

In the current study, the proportion of neoplasia in the head and neck region in dogs in Denmark was similar to other canine studies and significantly more common than in humans with a large proportion of malignancies. Spontaneous HNC in dogs thus, may serve as a model for HNC in humans.

Canine OMM is a spontaneous cancer in an outbred, immune-competent large mammal population and could be a clinical model for OMM in humans.

## Background

Cancer is the most common disease-associated cause of death or euthanasia in dogs [1-5]. Cancer is equally frequent in human medicine, where the age-standardised incidence rates in Denmark in 2004 were 697 and 605 per 100.000 for males and females, respectively [6].

Humans and dogs share a common environment and cancer in dogs has been proposed as a model of cancer in man [7-12]. It has been argued that the dog may represent a valuable model of cancer in humans and work as a sentinel of environmental carcinogens [12-14]. Dogs experience neoplasias to the same extent as humans despite their relatively shorter life span which may allow for accelerated investigations of environmental influences in the disease aetiology. Previous studies have shown that dogs react to environmental carcinogens in the same manner as humans. An association between environmental tobacco smoke and increased incidence of cancer in the nasal cavity and paranasal sinuses has been ascertained [11,15]. In addition, dogs in urban zones have been shown to be at risk of tonsillar carcinoma compared to dogs in rural zones [10]. Furthermore, in some cases, higher frequency of specific neoplastic diseases in dogs compared to humans offers an opportunity readily to investigate types of neoplasia rarely seen in humans. Dogs constitute a potentially more relevant clinical model with spontaneous neoplasms occurring in an outbred immuno-competent large mammal compared to the traditional experimental animal setting [13]. Spontaneous cancers in dogs, such as osteosarcomas and transitional cell carcinomas, have already been recognised to share traits like biological behaviour and therapeutic response with their human counterparts [12,16-18].

Head and neck cancers (HNC) are relatively common and often very serious diseases in both dogs and humans [6,19]. The incidence of human HNC in Denmark has increased during the last 20 years [6]. According to data from The Danish Cancer Registry; HNC represented 1178 (3.4%) out of a total of 35052 new cancer cases diagnosed in 2004 [6] while Hoffman *et al. *[20] reported that HNC accounted for 6.6% of cases reported to the National Cancer Data Base in the United States. Neoplasias originating in the head and neck region constitute a heterogeneous group with various anatomical locations and a wide spectrum of histopathological diagnoses. Regardless of this, HNC in humans are typically approached as one group partly due to the similar therapeutic approach in these tumours.

HNC often has an unfavourable prognosis due to the high proportion of malignant and invasive neoplasms. The proximity of the tissue structures renders extirpation of tumours with sufficient margins almost incompatible with preservation of functionality [21]. In humans oral malignant melanoma (OMM) represents a particular challenge since it is highly aggressive. Canine OMM is more frequent but share similar characteristics with human OMM and may thus be of interest as a spontaneous animal model [13,22]. Surgery is the treatment of choice in most of the neoplasms seen in HNC, but radiotherapy, chemotherapy and immunotherapy are also used when appropriate. Vital structures make radiation therapy (RT) difficult and treatment includes the risk of irradiation mucositis [23]. Chemotherapy is effective only against very few HNC, such as lymphoma [19]. Recent studies of immunotherapy have shown promising results in the treatment of canine OMM [13,22].

The objective of the current study was to describe the occurrence, biological behaviour, location, type and treatment of HNC in dogs registered in the Danish Veterinary Cancer Registry (DVCR). Furthermore the findings were compared to other registry studies in dogs and humans and the use of HNC in dogs as a model for HNC in humans was discussed, with special focus on OMM.

## Materials and methods

Canine cases entered in the DVCR from May 15^th ^2005 through February 29^th ^2008 were included in this study. The DVCR is a database of cases of neoplasia in Danish dogs and cats. It is an incident registry where each neoplasm is regarded as a separate entity, and data are collected prospectively. In contrast to other veterinary and human cancer registries DVCR comprises both benign and malignant neoplasms, and neoplasms diagnosed using other diagnostic methods than histology, such as cytology, diagnostic imaging etc.

Data were submitted using a web based submission form with an interface consisting of a questionnaire, in which veterinarians supplied data regarding the animal (i.e. age, gender, breed, postal code) and the neoplasm (i.e. type, behaviour, location, diagnostic approach). Cases were submitted from both small primary clinics and large referral hospitals and from clinics both from the capital region, larger cities as well as from rural areas of Denmark. The therapy utilised and cancer related euthanasia was also registered. The registration form was created based on the human cancer registry interface in order to facilitate later comparison and most variables are entered using drop-down menus for instant coding of data. Multiple neoplasms in a single individual were reported separately or manually separated if reported in bulk before evaluation took place.

### Inclusion criteria

Following the classification of HNC in the Danish Human Cancer registry (ICD 10) [6,24], cases of neoplasia submitted to DVCR located in the eyelid, oral cavity, oro- and nasopharynx, lip, tongue, nasal and sinus cavities, ear, salivary or thyroid gland were included in the current study. Cases of neoplasia with neoplasms located in the eye, brain and skin of the head and neck including the pinna were excluded.

Data was entered into an excel spreadsheet (Microsoft Office Excel) and statistical tests performed in SAS vs. 9.1 (SAS Institute, Cary, NC, USA). Fisher's exact test was used to compare proportions of HNC in dogs and humans as well as evaluation of the proportions of surgically treated cases of OMM and squamous cell carcinomas (SCC), the proportion of humans and dogs that were treated with surgery of all HNC. Also the proportions of benign and malignant in different locations of neoplasms in dogs were compared using Fisher's exact test.

## Results

During the study period a total of 1768 cases of neoplasia (679 malignant, 826 benign, 263 unknown) were submitted to the DVCR. Head and neck cancer accounted for 7.2% (n = 128) of these. Of the 128 HNC cases, 64 (50%) were malignant and 44 (34%) benign. In 20 (18%) cases no behaviour was submitted. If only malignant neoplasm were regarded, 9.3% of the total number of neoplasms was HNC. Sixty-eight neoplasms came from males (62 entire, 6 neutered), whereas 60 came from females (43 entire, 17 neutered) resulting in a male to female ratio (M:F) of 1.13, not only when considering all neoplasms, but also if only malignant neoplasms were included. The most common locations of HNC were the oral cavity, eyelids, the nasal cavity, lips and the thyroid gland which together accounted for 92% of the total number of HNC in the DVCR (Table 1). The majority of HNC were located in the oral cavity (46%) and 51% of these were malignant (Table 1). There was a significantly higher proportion of malignant than benign neoplasms in the nasal cavity and the thyroid gland compared to any of the other locations. In contrast there were significantly more benign neoplasms located in the eyelid than in other of the locations (*P *< 0.001).

**Table 1:** Anatomical location and biological behaviour of head and neck cancer in Danish dogs.

	Benign No (pct across)	Malignant No (pct across)	Unknown No (pct across)	Total No (pct of total)
Oral cavity	17 (29%)	30 (51%)	12 (20%)	59 (46%)
Eyelid	17 (94%)	1 (6%)	2 (11%)	18 (14%)
Nasal cavity	0 (0%)	16 (89%)	2 (11%)	18 (14%)
Lip	6 (40%)	8 (53%)	1 (7%)	15 (12%)
Thyroid gland	0 (0%)	5 (63%)	3 (38%)	8 (6%)

The most common types of malignant neoplasia were SCC (18; 28% of malignant), OMM (13; 20% of malignant), soft tissue sarcoma (11; 17% of malignant) and adenocarcinoma (5; 11% of malignant). The most common types of benign neoplasms were adenoma (7; 16% of benign), polyps (6; 14% of benign) and fibroma (5; 11% of benign) (Table 2).

**Table 2:** Most common malignant and benign neoplasms in the head and neck region of dogs.

Malignant neoplasms	Number (pct of total)	Pct. of total malignant or benign
Squamous cell carcinoma	18 (14%)	28%
Melanoma	13 (10%)	20%
Soft tissue sarcoma	11 (9%)	17%
Adenocarcinoma	5 (4%)	11%

**Benign neoplasms**		

Adenoma, sebaceous	7 (5%)	16%
Polyps	6 (5%)	14%
Fibroma	5 (4%)	11%
Histiocytoma	3 (2%)	7%
Papilloma	3 (2%)	7%
Epulis	3 (2%)	7%

Surgery was the most commonly used treatment of the HNC in dogs and was used in 74 (58%) of all cases (Table 3). Of these, two dogs had adjunctive therapy. One dog was treated with corticosteroids and one was treated with chemotherapy. Three dogs received only medical therapy in the form of corticosteroids. Dogs with OMM were treated with surgery in 11 cases (73%), whereas dogs with SCC were treated with surgery in only 3 cases (14%). Euthanasia was chosen in 30 cases of this study, two following surgery. Twenty-four of the 30 euthanised dogs had malignant neoplasms, which equals 38% of the dogs with malignant neoplasms.

**Table 3:** Verification of neoplasm and treatment for the different groups of biological behaviour

Verification	Surgery	Benign	Malignant	Uknown
Cytology	No surgery	2	19	4
	Surgery	5	5	4
Histology	No surgery	1	20	4
	Surgery	35	19	5
Other	No surgery	1	1	2
	Surgery	0	0	1

Total (pct surgery)	128 (58%)	44 (91%)	64 (38%)	20 (50%)

### Oral malignant melanoma

OMM was the second most common neoplasm in the head and neck region. Metastases were found in 5 out of 13 (38%) cases. Surgery was performed significantly more often in OMM cases than in cases of SCC (*P *< 0.0001). Chemotherapy was not used in any OMM cases. In 4 cases, euthanasia was chosen, one of these following surgery. Table 4 offers an overview of the OMM cases including information about grade if known, metastases and treatment, all data were extracted directly from the DVCR.

**Table 4:** Overview of 13 cases of oral malignant melanoma including grade if provided, metastases and if surgery was performed.

Case	Grade	Regional lymph node metastases	Distant metastases	Surgery performed
1	II	No	No	Yes
2	III	Yes	No	Yes
3	III	Yes	Yes	Yes
4	Unknown	No	No	Yes
5	Unknown	No	Unknown	Yes
6	Unknown	No	Unknown	Yes
7	Unknown	No	Unknown	Yes
8	Unknown	No	Unknown	No
9	Unknown	Yes	No	No
10	Unknown	Yes	No	Yes
11	Unknown	Unknown	No	Yes
12	Unknown	Unknown	Unknown	No
13	Unknown	Unknown	Unknown	Yes

## Discussion

The overall occurrence of HNC in the DVCR was 7.2% and if only malignant neoplasms were regarded, 9.3%. The malignant proportion was higher than in previous veterinary reports (4.2 to 6.3%) [25-29] as well as reports in humans (3.4 to 6.6%) [6,20,30]. The gender distribution was more equal in the current study than seen in other veterinary and human studies (M:F ratios from 1.50 to1.71) where males were more frequently represented than females [6,20,25,31]. In the current study, the proportion of malignant neoplasms (50%) was higher than in a Norwegian veterinary study (34%) [25]. The lower degree of malignancy in Norwegian HNC was due to a low proportion of malignancies in neoplasms of the oral cavity (31%). No other large studies included benign neoplasms, thus comparative data are scarce. The inclusion criteria of the DVCR comprising histopathological, cytological and clinical diagnostics and both benign and malignant neoplasms, contribute to the relatively high proportions in this study. Comparisons of various cancer registries thus, need to take variations in inclusion criteria into consideration. The DVCR is a prospective incidence registry based on record entries from all cases of neoplasms from all animals in a defined population. The DVCR has been validated against medical records with a high proportion (>95%) of agreement of key variables [32].

Similar to the present study, previous veterinary studies have reported the oral cavity as the most common location of HNC, accounting for 69% to 88% of the total HNC cases [24,26]. In humans the most prevalent sites include the oral cavity (18 to 24.6% of the cases) and larynx (20.8% to 28.5%) [6,20,31]. In the current study no cases were located in the larynx. Cancers of the larynx in humans are strongly associated with repeated exposure to cigarette smoke and alcohol [33,34]. The nasal cavity accounted for more cases in the current study (14%) compared to other veterinary (4.4 to 6.1%) as well as human studies (5.1%) [6,25,26]. A previous study showed a connection between increased prevalence of nasal tumours and passive smoking in dogs [10].

More than half of the HNC cases (55.8%) in humans were SCC originating in the larynx and the oral cavity. Other common types of HNC reported in humans are adenocarcinomas and lymphomas [20,31]. In humans the most common types of malignant neoplasms were SCC, adenocarcinoma and lymphoma [20]. Squamous cell carcinoma was predominant in both dogs and humans, but soft tissue sarcoma and malignant melanoma were more frequent in dogs than humans.

A standardised treatment scheme is not available for all types of HNC, but surgery remains the primary therapeutic modality in dogs in Denmark. However adjunctive treatment e.g. RT is increasingly being added to the treatment protocols [35]. In humans, RT is more widely utilised, 25.0 to 26.8% of the cases in humans were treated with both surgery and RT, and 18.9 to 29.5% were treated with RT alone, while surgery as a sole treatment was used in 32.4 to 34.2% of the cases [20,31]. RT was not used in any of the cases in the current study. This type of therapy is at this point not readily available to veterinary patients in Denmark, and when available very few owners choose to take advantage of this modality.

### OMM as an example of dogs serving as models for OMM in humans

Studies into aetiology, risk factors and treatment response may be conducted faster and include more individuals if performed in dogs instead of people. Spontaneously occurring types of neoplasia commonly found in dogs but rarely in humans have the potential to be exerted as models of cancer in humans. A phenotypically well characterised highly prevalent spontaneous canine model for rare human cancers opens the possibility for in vivo research with a short interval between generations.

The proportion of HNC of all tumours in dogs in DVCR was significantly higher than the proportion of humans with HNC in Denmark (*P *< 0.0001) [6] which was also evident when compared to other studies [36,37]. In dogs SCC and OMM were the most commonly reported types of HNC both in the current and other veterinary studies [38,39]. OMM accounted for 20% to 50% of oral malignancies in other veterinary studies, similar to the current study [13,27,40]. However in humans, primary OMM is extremely rare [20,36,41] and often bears a poor prognosis with a 5 year survival of 12.3% to 17.2% and metastases are frequently observed [36,42]. Dogs equally have short life expectancies following a diagnosis of OMM and in one study distant metastasis were found at necropsy in 47% to 67% of the OMM cases, consistent with the current study [38].

The similarities in biological behaviour, treatment response and severity of canine OMM suggest dogs could make an interesting and relevant model for OMM in humans [43]. Spontaneous canine cancers may serve as an important bridge between preclinical studies in mouse model systems and clinical trials in humans with cancer and support the synergy of collaborations between veterinary and human cancer centers [22]. Such collaboration has already resulted in the development of an immunotherapy in dogs with grade II OMM and has shown promising results [44]. This type of treatment is also being investigated in humans [45,46]. The use of spontaneously developing cancer in the companion animal population could potentially lower the number of experimental animals needed in research, in line with the Three R's concept (Replacement, Reduction, Refinement) [47]. Furthermore dogs provide a more faithful preclinical therapeutic model compared to the traditional mouse systems as both biological behaviour and response to treatment are similar to those seen in humans [22,43]. And importantly, dogs may also benefit from being models as the latest treatment options would be made available to them in a clinical trial setting.

## Conclusions

In the current study the proportion of neoplasia in the head and neck region in dogs in Denmark was similar to other canine studies and significantly more common than in humans, with a large proportion of malignancies, and a slight overweight of males.

This suggests that spontaneous HNC in dogs may serve as a model for HNC in humans. Canine OMM would be a more faithful preclinical model for OMM in humans compared with the more traditional mouse systems. OMM in canines is a spontaneous cancer in an outbred, immune-competent large mammal population that shares the environment of humans.

## Competing interests

The authors declare that they have no competing interests.

## Authors' contributions

LBB carried out data management and statistical analysis, participated in designing thestudy, evaluating results, researching background literature and drafting the manuscript. TE participated in designing the study, evaluating results, researching background literature and drafting the manuscript. ATK supplied the foundation and idea for the study as well as participated in coordination and drafting the manuscript. All authors read and approved the final manuscript.
